# Differences in Tumor-Associated Protein Levels among Middle-Age Flemish Women in Association with Area of Residence and Exposure to Pollutants

**DOI:** 10.1289/ehp.8811

**Published:** 2006-02-09

**Authors:** Nicolas A. van Larebeke, Marc E. Bracke, Vera Nelen, Gudrun Koppen, Greet Schoeters, Herman Van Loon, Robert Vlietinck

**Affiliations:** 1 Study Centre for Carcinogenesis and Primary Prevention of Cancer and; 2 Laboratory of Experimental Cancerology, Department of Radiotherapy, Nuclear Medicine, and Experimental Cancerology, Ghent University Hospital, Ghent, Belgium; 3 Provincial Institute for Hygiene, Antwerp, Belgium; 4 Centre of Expertise in Environmental Toxicology, Flemish Institute of Technological Research, Mol, Belgium; 5 Genetic Epidemiology, Center of Human Genetics, Katholieke Universiteit, Leuven, Belgium

**Keywords:** anti-p53, biomonitoring, bladder tumor antigen, CA 125, cancer, dioxin-like activity, environment, lead, tumor markers, zinc

## Abstract

We measured tumor-associated proteins (TAPs) and pollutants in blood, serum, and urine of 200 nonsmoking women 50–65 years of age, residing in the rural municipality of Peer or in Hoboken or Wilrijk, industrial suburbs of Antwerp, Belgium. Persons with occupational exposures or commuting to other towns were excluded. Residents from Hoboken had significantly higher levels of blood lead and serum zinc and polychlorinated biphenyls. Surprisingly, residents of Peer had significantly higher levels of serum cadmium, dioxin-like activity in blood fat, and urinary 1-hydroxypyrene. For 5 of the 12 TAPs assessed in this study, we observed significant differences in serum levels among residents of the three municipalities after adjusting for personal or lifestyle parameters. Although we found levels of internal exposure to pollutants to be quite homogeneous in Flanders, we found significantly higher levels of TAPs only in the industrial suburbs. In multiple regression with all 29 available personal, lifestyle, and internal exposure parameters, blood lead levels showed a positive association with serum levels of anti-p53, carcino-embryonic antigen (CEA), and tissue polypeptide–specific antigen (TPS) and with an index for mean TAP level (*I*_tap_); dioxin-like activity in serum and serum copper showed a positive association with serum CA 125 (cancer antigen 125); and serum zinc showed a positive association with serum levels of c-erbB-2 ectodomain and TPS. An index of internal exposure showed a positive association with serum levels of both CEA and anti-p53 and with *I*_tap_. This study provides some evidence that levels of internal exposure such as those present in Flanders, in particular concerning lead, are indeed associated with biologic effects.

The Flemish Environment and Health Study (FLEHS) evaluated whether residence in areas with different types and concentrations of environmental pollutants had a significant impact on internal exposure to pollutants and whether place of residence or observed differences in internal pollutant concentrations were associated with biologic and health effects. We measured both internal exposure and effect biomarkers on an individual basis, in residents of Wilrijk and Hoboken, industrial suburbs of the city of Antwerp, Belgium, and of Peer, a rural municipality in Flanders, the Dutch-speaking northern part of Belgium. Characteristics of the study areas have been described previously (van Larebeke et al. 2004). Results concerning adolescents from the same areas have been published (Nawrot et al. 2002; Staessen et al. 2001; Van Den Heuvel et al. 2002).

During the long latency period after initiation of carcinogenesis and/or under the impact of tumor promoters, some cells in the body might express certain aspects of the tumoral phenotype, which may result in macromolecules associated with this phenotype being released into body fluids. An increased concentration of such molecules in body fluids might, to some extent, reflect a higher exposure to cancer-inducing or cancer-promoting agents (Koshida et al. 1990; Krajewska et al. 1998; Luo et al. 1999; Lutz et al. 1997; Wong et al. 2002) or an increased risk of cancer (Brandt-Rauf and Pincus 1998; Giovannuci 1999; Hankinson et al. 1998; Jacobs et al. 1996; Jeyarajah et al. 1999; Kobayashi and Kawakubo 1994; Oka et al. 1994; Wolk et al. 1998). At the beginning of our study, we hypothesized that differences in exposure to pollutants between the three study areas might have led, directly or through diverse cancer-related biologic processes, to differences in tumor-associated protein (TAP) levels. In this article we report on TAP levels in serum or urine of 200 nonsmoking women 50–65 years of age (100 from Peer, 39 from Wilrijk, and 61 from Hoboken). Possible confounders related to lifestyle and personal characteristics were taken into account.

Results concerning dioxins and polychlorinated biphenyls (PCBs) on adults in the FLEHS have already been published (Covaci et al. 2001; Koppen et al. 2001). We previously (van Larebeke et al. 2004) reported data on hypoxanthine phosphoribosyltransferase (*HPRT*) gene mutant frequency (*HPRT*_mf_) in a subset of the subjects participating in the present study.

## Materials and Methods

### Selection of study areas

Hoboken and Wilrijk are located 11–13 km southeast of the chemical and petrochemical industry established in the seaport of Antwerp. Hoboken harbors a large nonferrous smelter (the cause of important lead pollution in the past), other metallurgic industries, a large printer, and plants producing electronic equipment. The mean lead concentration of suspended particles during 1997 in Hoboken was between 0.08 and 1.35 μg/m^3^, depending on the location. Wilrijk contains metallurgic industry, plastic industry, a printer, a crematorium, and two waste incinerators with annual turnovers of 23,000 and 110,000 tons; these incinerators were shut down in November 1997 because of dioxin emissions exceeding the acceptable level [0.1 ng 2,3,7,8-tetrachlorodibenzodioxin (TCDD) toxic equivalents (TEQ)/m^3^] by 20-fold. Both Hoboken and Wilrijk are traversed by a highway traveled by > 80,000 vehicles a day. Peer (14,622 inhabitants), an area of intensive agriculture, is situated in a rural area 70 km east of Antwerp. Although there is a military airfield about 5 km north of the municipality and some polluting industries about 15 km away, measured environmental pollution was lower in Peer than in many other places in Flanders. Sources of and data on environmental pollution of these municipalities were described previously (van Larebeke et al. 2004).

### Selection of the study population

We chose middle-age women because they spent most of their lives residing in the same region and working at home. We measured TAP levels in serum samples from 200 women from Hoboken (*n* = 61), Wilrijk (*n* = 39), and Peer (*n* = 100) recruited between June and September 1999. To obtain the planned 200 women for the study, we used the following selection strategy. We contacted (only one attempt) all female residents 50–65 years of age (2,898 women) by letter containing a questionnaire on age, duration of residence, smoking, occupation and place of occupation, number of children, consumption of vegetables or fruit from the family’s garden, and vegetarianism. In Hoboken/Wilrijk and Peer, 40.1% and 30.8% responded, respectively. We used the following two inclusion criteria: minimal residence of 10 years in the study area and employment in the town of residence or at home for at least 10 years. We excluded smokers and persons who stopped smoking < 10 years before the start of the study as well as persons who were ever employed in jobs with specific risks of exposure. Having been diagnosed with cancer or another disease was not an exclusion criterion. We ranked the remaining 685 women by duration of residence and smoking history, with never-smokers given priority and ex-smokers prioritized by time elapsed since cessation of smoking. In order to reach 100 participants per region, we used telephone calls to invite 130 women from Wilrijk or Hoboken and 125 women from Peer to participate in the study. Of the 130 residents from Wilrijk or Hoboken, 12 refused to participate and 18 others did not participate for other reasons. Of the 125 residents from Peer, 14 refused to participate and 11 others did not participate for other reasons. We noted no significant differences between participants and nonparticipants in terms of their responses to items in the questionnaire.

All participants gave their written informed consent. Throughout the study, a communication plan (with the participants and local authorities) was in force. Each participant was offered the possibility of having an interview with a physician-researcher. We complied with all applicable requirements. The study was approved by the institutional review board of the University of Antwerp.

### Measured parameters

We measured height and body weight, calculated body mass index (BMI), and collected approximately 200 mL urine and 40 mL blood from each subject. Blood samples were collected in polyethylene tubes. Immediately after sampling, serum was separated. Split samples of serum, plasma, whole blood, and urine were stored at 4°C or immediately deep frozen. All laboratory analyses were performed blindly in specialized laboratories that met national and international quality-control standards. For each participant, we performed a series of routine hematologic tests and determined serum concentrations of total fat, triglycerides, cholesterol, selenium, vitamin A, vitamin E, zinc, and copper. We measured the following biomarkers of exposure in serum, blood, or urine as described previously (van Larebeke et al. 2004): dioxin-like activity, expressed in picograms TEQ per gram blood fat; PCB-138, PCB-153, and PCB-180 (indicator PCBs), expressed in nanograms per gram blood fat [we calculated the sum of the concentrations of these PCBs (∑3PCBs) to use as a parameter in statistical analysis]; cadmium in urine and blood; lead in blood; and 1-hydroxypyrene in urine. TAP levels in urine [bladder tumor antigen (BTA)] or serum (all other parameters) were measured by Interlab (Evergem, Belgium), a laboratory recognized by the Belgian Ministry of Health as a reference laboratory for tumor markers. We measured the following TAPs: tissue polypeptide–specific antigen (TPS; TPS IRMA kit; BEKI Diagnostics, Bromma, Sweden); human placental alkaline phosphatase (hPLAP; Innotest hPLAP kit; Innogenetics, Ghent, Belgium); c-erbB-2 ectodomain (c-erbB-2; Human neu Quantitative ELISA kit; Oncogene Research Products, Cambridge, MA, USA); insulin-like growth factor-I (IGF-I; IGF-I ELISA, DSL-10-600 kit; DSL, Webster, TX, USA); platelet-derived growth factor BB (PDGF-BB; Quantikine Human PDGF-BB kit; R&D Systems, Minneapolis, MN, USA); carcino-embryonic antigen (CEA; IRMA-coat CEA kit; Byk-Sangtec Diagnostica, Dietzenbach, Germany); cancer antigen (CA) 15-3 (IRMA-count BR-MA kit; DPC, Los Angeles CA, USA); CA 125 (IRMA-mat CA 125 II kit; DPC); α-fetoprotein (AFP; COAT-A-COUNT AFP IRMA kit; DPC); BTA (BTA TRAK kit; Bard Diagnostic Sciences, Redmond, WA, USA); anti-p53 (Anti-p53 ELISA kit; PharmaCell, Paris, France); soluble interleukin-2 receptor (sIL-2R; Milenia IL2R kit; DPC). Except for BTA, urinary measurements were standardized to 1 mmol creatinine. Coefficients of variation of the TAPs in the clinically normal range in our laboratory (expressed as percentage) were 5.0% for anti-p53, 5.0% for c-erbB-2, 8.7% for TPS, 7.1% for AFP, 9.8% for CEA, 6.5% for CA 15-3, 11.0% for CA 125, not available for hPLAP, 4.7% for BTA, 7.1% for IGF-I, 8.5% for sIL-2R, and 7.7% for PDGF-BB.

### Parameters resulting from questionnaires

All participants filled out a self-reporting questionnaire on education, residence history, food intake, smoking, alcohol habits, and health status. We classified education by the highest education received: primary school or the first 3 years of secondary school, complete secondary school, and higher education. We considered participants ex-smokers if they had smoked at least one cigarette per day for at least 1 year; participants who smoked less than this were classified as never-smokers. We used the following food-intake parameters, calculated from the questionnaires on dietary habits over the last year: ethanol in grams per day, total daily intake of animal fat in grams, frequency of consumption of dairy products per day, total daily intake of calcium in milligrams, and number of different types of locally grown food items regularly consumed. Also, we recorded frequencies of monthly consumption of meat (smoked, salted, grilled, roasted, baked, or organ meat); baked, roasted, or grilled meat; fish; smoked or salted meat or fish; fish, fish organ meat, mussels or shrimp; and vegetables from own garden. In addition, we calculated the total number of pregnancies and the total number of weeks of breast-feeding. We made no attempt to collect information on occurrence of cancer in family members.

### Statistical analyses

For each TAP and for each biomarker of exposure we calculated a standard or *z*-score for each individual by dividing the difference between the value for that individual and the mean value for the entire subject population by the standard deviation for the entire subject population. We calculated for each individual an index of mean TAP level (*I*_tap_), defined as the arithmetic mean of the *z*-scores for each type of TAP [*I*_tap_ = (*z*_TPS_ + *z*_hPLAP_ + *z*_c-erbB-2_ + *z*_IGF-I_ + *z*_PDGF-BB_ + *z*_CEA_ + *z*_CA 15-3_ + *z*_CA 125_ + *z*_AFP_ + *z*_BTA_ + *z*_anti-p53_ + *z*_sIL-2R_)/12]. For each subject we calculated an index of internal exposure (*I*_ex_), defined as the arithmetic mean of the *z*-scores for blood lead concentration; ∑3PCBs; dioxin-like activity in serum measured by the chemically activated luciferase expression (CALUX) bioassay; urinary excretion of cadmium per millimole creatinine; and urinary excretion of 1-hydroxypyrene per millimole creatinine [*I*_ex_ = (*z*_blood lead_ + *z*_∑3PCBs_ + *z*_dioxin-like activity_ + z_urinary cadmium_ + z_urinary 1-hydroxypyrene_)/5]. We performed stepwise regression, multiple regression, analysis of covariance (ANCOVA), and accompanying Fisher’s probable least-squares difference (PLSD) post hoc tests, chi-square, non-parametric Spearman rank correlation, Mann-Whitney *U*, and Kruskal-Wallis tests using Statview (version 5.0.1; SAS Institute, Cary, NC, USA) or Statistica (Statsoft, Tulsa, OK, USA) programs. We transformed parameters that did not show a Gaussian distribution for use as dependent variables in an ANCOVA analysis; we used the natural logarithms of the serum concentrations of anti-p53, TPS, AFP, CA 15-3, CA 125, BTA, IGF-I, and sIL-2R and the square root of the concentrations of c-erbB-2, CEA, hPLAP, and PDGF-BB. To select parameters associated with differences in TAP levels for use in ANCOVA analysis or in multiple regression, we performed a forward stepwise regression of personal characteristics and lifestyle parameters (except those related to locally grown food) for ANCOVA analysis and personal, lifestyle, and internal exposure parameters for use in multiple regression, with each TAP as a dependent variable using *F* = 4 for inclusion and *F* = 3.9 for exclusion. Adjustment through ANCOVA analysis resulted in geometric mean values. ANCOVA analyses were limited to main effects only. In accordance with the views formulated by Rothman (1986), we present *p*-values without adjustment for multiple testing. However, to address concerns regarding multiple testing, we also provide the *p*-values required to maintain an overall type I error bound of 0.05 after adjustment for multiple comparisons according to Holm (Aickin and Gensler 1996). In relation to ANCOVA and multiple regression analyses, we calculated *p*-values taking into account that 12 tests—one for each TAP—were performed. Results from multiple regression include the squared semipartial correlation, which is a measure of the correlation between two variables that remains after controlling for the effects of the other predictor variables and indicates the proportion of variance accounted for by the predictor *X*_1_ relative to the total variance of *Y*.

## Results

### Internal exposure

Table 1 shows internal exposure data (concentrations in blood, serum, and urine). We found that residents from Hoboken had significantly higher blood lead, serum zinc, and serum PCB levels than residents from other municipalities. Also, compared with residents from the other municipalities, residents from Peer had significantly higher serum cadmium levels, higher dioxin-like activity in blood fat (significantly so compared with Wilrijk), and higher 1-hydroxypyrene levels (significantly so compared with Hoboken).

### Levels of TAPs in sera from residents of different areas

Crude data are summarized in Table 2. Depending on the type of TAP, between 91.4% and 100% of participants showed clinically normal values, except with respect to BTA, with only 80.7% normal values. ANCOVA revealed that place of residence was significantly associated with differences in c-erbB-2, BTA, and PDGF-BB levels and, after correction for multiple comparisons, with differences in BTA and PDGF-BB levels. Using a Fisher PLSD test, we found significantly higher TAP levels for both industrial suburbs but not for the rural municipality of Peer. Women residing in Hoboken had the highest levels of c-erbB-2, TPS, and hPLAP. For c-erbB-2, the difference was significant for both Peer (*p* = 0.0099) and Wilrijk (*p* = 0.026); for TPS (*p* = 0.049) and hPLAP (*p* = 0.015), the difference was significant only for Wilrijk. Although residents from Hoboken had lower PDGF-BB levels than those from Wilrijk, their levels were significantly higher than those of residents from Peer (*p* = 0.0021). Residents of Wilrijk showed significantly higher levels of BTA than those of Hoboken (*p* = 0.0006) and those of Peer (*p* < 0.0001) and significantly higher PDGF-BB levels than those of Hoboken (*p* = 0.023) and those of Peer (*p* < 0.0001).

Adjustment for potentially confounding personal and lifestyle parameters (age; level of education; smoking status; total number of pregnancies; total number of months of breast-feeding; BMI; serum levels of selenium, vitamin A, vitamin E, total fat, total cholesterol, and triglycerides; and all available parameters related to food intake except those related to consumption of locally grown food, a total of 21 parameters) had no substantial effect on the association of TAP levels with area of residence (Table 3). Results of ANCOVA were similar, except that area of residence was now significantly associated with differences in hPLAP levels but no longer with differences in c-erbB-2 levels. Using the Fisher PLSD test, the observed differences between residents of the different area’s remained significant.

The *I*_tap_, both without correction (data not shown) and after correction for place of residence, all available personal and lifestyle parameters, and all available food-intake parameters, was higher in residents of Hoboken (adjusted value = 0.074) than in residents of Wilrijk (−0.020) or Peer (−0.040), and the difference with Peer was close to statistical significance (*p* = 0.058 after correction for confounding).

### Association of TAP levels with levels of biomarkers of internal exposure for the whole study population

We used the levels of TAPs as dependent variables and all 29 available personal, lifestyle, and internal exposure parameters as independent variables in multiple regression analysis (Table 4). We found a positive correlation of blood lead with serum levels of anti-p53, CEA, and TPS and with *I*_tap_; a positive correlation of serum zinc with c-erbB-2 and with TPS; a positive correlation of dioxin-like activity in serum with CA 125; and a negative correlation of serum copper with IGF-I and c-erbB-2. The *I*_ex_ showed a positive association with serum levels of CEA and of anti-p53.

If the total study population was divided into two classes according to the *I*_ex_ [women with an *I*_ex_ higher than the median versus the others, both without correction (data not shown)] and after correction for place of residence, all available personal and lifestyle parameters, and all available food-intake parameters, the class with higher *I*_ex_ levels had significantly higher values for *I*_tap_ (adjusted mean, +0.075 vs. −0.078; *p* = 0.010).

### *Association of TAP levels with* HPRT*_mf_*

FLEHS entailed measurements of *HPRT*_mf_ in peripheral lymphocytes for a subset of the women who participated in the study (van Larebeke et al. 2004). For 11 of the 12 TAPs and for *I*_tap_, we found a nonsignificant positive association with *HPRT*_m f_, whereas for PDGF-BB we found a non-significant negative association (chi-square, *p* = 0.004). In multiple regression with all available personal, lifestyle parameters, and internal exposure parameters, CEA showed a significant positive correlation with *HPRT*_mf_ (squared partial correlation = 0.108, *p* = 0.018).

## Discussion

### Selection bias

Although possible, it is unlikely that a selection bias occurred regarding participation in Wilrijk, Hoboken, and Peer. The quite low response rate may be because only one letter was sent and participants were required to give blood and urine; only 12 of 130 respondents from Wilrijk or Hoboken and only 14 of 125 from Peer actually refused to participate. Concerning a series of items on which respondents provided information (described in “Materials and Methods”), we found no significant differences between participants and persons who refused or could not participate.

### Disease and genetic constitution

The study participants generally considered themselves to be in good health (data not shown). We considered it preferable not to exclude persons presenting with complaints or with a disease, in order to avoid introducing a bias by eliminating persons whose illness or complaints might be influenced by environmental factors. Because only a limited percentage of cancer cases can be ascribed to hereditary factors (Czene et al. 2002; Verkasalo et al. 1999), because the populations in the areas under study are genetically probably quite similar (distances are small, the landscape permits easy traveling, no historical divisions, all participants spoke the same language), and because selection bias is unlikely, we consider it improbable that the observed differences in TAP levels as a function of area of residence or of internal exposure are due to hereditary factors.

### Differences in internal exposure

Residents from Hoboken had significantly higher blood lead and serum zinc levels than residents from other municipalities, which was to be expected in view of the presence of a large nonferrous smelter; they also showed significantly higher serum PCB levels, which was less expected. Surprisingly, however, compared with residents from the other municipalities, residents of the rural municipality of Peer had higher serum cadmium levels, higher dioxin-like activity in blood fat (significantly so compared with Wilrijk), and higher 1-hydroxypyrene levels (significantly so compared with Hoboken). We are not aware of a reliable explanation of these high internal exposure levels in residents of Peer; some of them may have originated from previous exposures (cadmium, dioxin-like activity). Also it remains possible that both intensive agriculture (pesticides, fertilizers) and some industrial activities at a distance of about 15 km were involved. In 2000–2001, episodes of air pollution with polycyclic aromatic hydrocarbons and high mutagenic activity were observed in Peer (Du Four et al. 2004). These data and also the data from an ongoing Flemish biomonitoring program involving other rural areas (Milieu en Gezondheid 2006) indicate that pollution is widespread in Flanders and suggest that exposure to pollutants may be quite homogeneous in some developed Western nations.

### Meaning of differences in levels of TAPs

Whether differences in levels of TAPs, within clinically normal ranges, are of importance in terms of health risks is not known with certainty. There are, however, a series of observations suggesting that exposure to carcinogenic or tumor-promoting agents leads to a slight increase in these levels, including in those of TAPs examined in this study (Koshida et al. 1990; Krajewska et al. 1998; Luo et al. 1999; Lutz et al. 1997; Wong et al. 2002). Also, in a number of studies, higher levels of TAPs, still in the clinically normal range, were associated with a higher risk of cancer. Such an association was clearly demonstrated for prostate-specific antigen and risk of prostate cancer (Ito et al. 2003, 2005) and has also been observed for the TAPs used in this study (Bohlke et al. 1998; Brandt-Rauf and Pincus 1998; Giovannuci 1999; Hankinson et al. 1998; Husgafvel-Pursiainen et al. 1997; Jacobs et al. 1996; Jeyarajah et al. 1999; Kobayashi and Kawakubo 1994; Oka et al. 1994; Wolk et al. 1998). The trend toward a positive association between levels of TAPs and *HPRT*_mf_ is consistent with the hypothesis that these parameters are biomarkers of exposure to carcinogenic agents or of cancer risk.

### Internal exposure and TAPs

For the whole study population, certain parameters of internal exposure known or suspected to be associated with an increased risk of cancer showed a positive association with the serum or urinary levels of some TAPs or with the *I*_tap_. There is ample experimental evidence that inorganic lead is carcinogenic (Silbergeld et al. 2000), and it is classified by the International Agency for Research on Cancer (IARC) as group 2B, possibly carcinogenic to humans (IARC 1987). In the general U.S. population, a dose–response relationship between quartile of blood lead and all cancer mortality showed relative risks of 1.24, 1.33, and 1.5 for the second, third, and fourth quartiles, respectively, compared with the first quartile, but this relationship was not significant (*p*-trend = 0.16) (Jemal et al. 2002). Workers exposed to lead have been reported to have an increased incidence of lung cancer (Anttila et al. 1995). In our study on women without occupational exposure and with blood lead levels within “normal” limits, blood lead showed a significant positive correlation with serum levels of anti-p53, CEA, and TPS and with *I*_tap_, this in multiple regression analyses with all available personal and lifestyle parameters and with all available parameters of internal exposure. In the same cohort, blood lead also showed a positive correlation with *HPRT*_mf_ (van Larebeke et al. 2004). In the vicinity of a nonferrous smelter in Sweden, an increased incidence of lung cancer in men has been found (Besso et al. 2003). The findings presented here and in our previous report (van Larebeke et al. 2004), taken together, suggest that lead could be an important carcinogen for at least part of the population included in our study.

Dioxins are human carcinogens associated with an increase in total cancer incidence in humans (IARC 1997) and with endometriosis in apes (Rier et al. 1993, 1995) and in women (Koninckx et al. 1994; Mayani et al. 1997); we found a positive association between dioxin-like activity in serum and serum CA 125. Serum CA 125 concentrations are often higher in women with endometriosis (Somigliana et al. 2004).

Zinc deficiency may enhance carcinogenesis, and zinc is under consideration as a chemopreventive agent (Riboli et al. 1996). However, zinc also has a genotoxic potential; it has induced cell transformation *in vitro*, and an oversupply of zinc may increase the risk of prostate cancer (European Commission 2003). In the present study, serum zinc level showed a positive correlation with c-erbB-2 and TPS.

The relationship between copper and the risk of cancer is complex (Theophanides et al. 2002). In our study, serum copper showed a weak but significant positive correlation with CA 125, but a quite strong negative correlation with c-erbB-2 and IGF-I levels.

The *I*_ex_, integrating parameters related to lead, cadmium, PCBs, dioxin-like activity, and 1-hydroxypyrene, was calculated for each participant as described in “Materials and Methods.” Although such an index does not take into account possible interactions, it might give a somewhat more integrated view on the internal exposure of an individual. The *I*_ex_ showed a positive association with *I*_tap_ and with serum levels of anti-p53 and CEA.

### Area of residence and TAPs

For 5 of the 12 TAPs assessed in this study (i.e., BTA, c-erbB-2, PDGF-BB, TPS, and hPLAP), we observed significant differences in serum levels between residents of the three municipalities. After correction for all 21 available personal or lifestyle parameters, the differences remained significant. For each of these five TAPs, we observed the highest levels in one of the industrial suburbs, although, compared with residents from the other municipalities, residents of the rural municipality of Peer had higher serum cadmium levels, higher dioxin-like activity in blood fat, and higher urinary 1-hydroxypyrene levels. Even when correction through ANCOVA analysis included all available internal exposure parameters in addition to all 21 available personal or lifestyle parameters, these differences remained significant (data not shown). This suggests that the higher levels of some TAPs in the industrial suburbs might, at least in part, be caused by exposures other than those measured in this study, or by other unknown factors somehow associated with residence in these suburbs.

## Conclusions

Although we found levels of internal exposure to pollutants to be quite homogeneous in Flanders, and although we observed the highest levels of some pollutants in the rural municipality of Peer, we found significantly higher levels of TAPs only in the industrial suburbs. This suggests that, in biomonitoring, effect biomarkers might indeed be important in addition to biomarkers of internal exposure. Also, this study provides some evidence indicating that levels of internal exposure such as those present in Flanders, lead in particular, are indeed associated with biologic effects and that even relatively small differences in these levels are associated with observable differences in such effects. Our observations suggest that more research into the use of TAPs to assess, in an integrated manner, the biologic effects of exposure to carcinogenic or tumor-promoting agents is of interest.

## Figures and Tables

**Table 1:** Internal exposure concentrations in blood, serum, and urine.

	Wilrijk^a^	Hoboken^a^	Peer	*p*-Value^b^
Blood or serum concentration				
Cadmium (nmol/L)	5.34 (3.56–11.21)*	5.34 (3.56–10.23)**	6.23 (3.56–11.57)	0.0097
Copper (μmol/L)	20.4 (15.1–26.6)	19.8 (16.3–25.4)	19.3 (16.5–25.8)	0.56
Lead (nmol/l)	154.4 (71.4–229.7)^##^	183.4 (108.6–337.8)**^,##^	152.0 (84.5–255.8)	0.0029
Zinc (μmol/L)	9.64 (8.17–10.89)^###^	10.40 (9.26–12.47)***^,###^	9.49 (7.65–11.41)	< 0.0001
Dioxin-like activity (pg TEQ/g blood fat)	30.8 (5.1–71.8)**	41.9 (6.4–81.3)	44.2 (16.6–80.3)	0.010
∑PCBs (ng/g blood fat)	379 (230–621)^#^	459 (270–675)***^,#^	370 (238–604)	0.0006
Selenium (μmol/L)	1.26 (1.04–1.53)***	1.21 (0.96–1.52)***	1.10 (0.88–1.31)	< 0.0001
Vitamin A (μmol/L)	2.06 (1.45–2.94)	2.06 (1.62–2.95)**	1.85 (1.43–2.57)	0.036
Vitamin E (μmol/L)	30.5 (23.2–38.6)	32.6 (25.5–46.7)	32.5 (25.6–45.9)	0.093
Concentration in urine
Cadmium (nmol/mmol creatinine)	0.73 (0.40–1.70)	0.74 (0.45–1.59)	0.97 (0.44–1.87)	0.43
1-Hydroxypyrene (pmol/mmol creatinine)	46.1 (7.5–92.9)	38.0 (16.6–84.9)*	49.0 (15.1–191.3)	0.026

Values shown are median (10th–90th percentile).

a Significant differences by Mann-Whitney *U* test between urban areas and Peer (*p ≤0.05, **p ≤0.01, and ***p ≤0.001), and between both urban areas (^#^*p* ≤0.05, ^##^*p* ≤0.01, and ^###^*p* ≤0.00).

b Kruskal-Wallis test for differences between areas; if correction for multiple testing is implemented, statistical significance requires *p* < 0.0063.

**Table 2:** Levels of TAPs: crude data.

TAP	Wilrijk^a^,^b^ (*n* = 39)	Hoboken^a^,^b^ (*n* = 61)	Peer (*n* = 100)	Normal values^c^	Residence ANCOVA *p*-value^b^
Anti-p53 (index)	0.318 (0.050–0.589)	0.464 (0.050–1.078)	0.433 (0.073–0.755)	0.0–1.1	0.16
c-erbB-2 (pmol/mL)	2.34 (1.73–2.82)^#^	2.46 (1.56–3.65)*^,#^	2.34 (1.72–2.82)	NA	0.020
TPS (U/L)	31.0 (20.4–40.8)^#^	34.0 (18.0–80.5)^#^	31.0 (21.0–55.8)	0–80	0.14
AFP (ng/mL)	1.50 (0.80–2.96)	1.55 (1.00–3.05)	1.60 (1.10–3.46)	0–20	0.37
CEA (ng/mL)	0.30 (0.30–1.58)	0.60 (0.30–2.45)	0.60 (0.30–1.60)	0–3.5	0.51
CA 15-3 (U/mL)	16.8 (11.8–25.1)	16.1 (9.4–28.2)	16.4 (10.2–25.6)	0–38	0.46
CA 125 (U/mL)	13.1 (5.7–25.5)	10.2 (5.6–25.1)	12.4 (5.9–31.0)	0–29	0.60
hPLAP (mU/L)	1.60 (0.10–28.96)^#^	14.75 (0.10–37.55)^#^	8.20 (0.10–29.95)	0–100	0.14
BTA (U/mL)	9.20 (1.72–31.52)***^,##^	3.50 (0.65–17.89)^##^	3.20 (0.65–18.50)	0–14	0.0001
IGF-I (ng/mL)	139 (88–260)	148 (91–241)	168 (102–231)	91–443	0.27
sIL-2R (U/mL)	385 (120–617)	344 (120–630)	385 (172–670)	85–961	0.26
PDGF-BB (pg/mL)	3,340 (1,792–5,532)***^,#^	2,550 (1,070–4,780)**^,#^	2,060 (280–3,534)	942–7,366	< 0.0001

NA, not available. Values shown are median (10th–90th percentile).

a Significant differences by Fisher’s PLSD test on crude data, between urban areas and Peer (*p < 0.01, **p < 0.0045, and ***p < 0.0001), and between Wilrijk and Hoboken (^#^*p* < 0.05, and ^##^*p* < 0.0045).

b If correction for multiple testing is implemented, statistical significance requires *p* ≤0.0045.

c As established by the manufacturer of the measuring kit and controlled on a sample of the Flemish population.

**Table 3:** Mean levels of TAPs adjusted for personal characteristics and lifestyle factors.

TAP	Wilrijk^a^,^b^ (*n* = 39)	Hoboken^a^,^b^ (*n* = 61)	Peer (*n* = 100)	Residence ANCOVA *p*-value b
Anti-p53 (index)	0.258	0.364	0.338	0.27
c-erbB-2 (pmol/mL)	2.36^#^	2.49*^,#^	2.28	0.098
TPS (U/L)	28.7^#^	38.9^#^	34.3	0.055
AFP (ng/mL)	1.59	1.63	1.78	0.51
CEA (ng/mL)	0.61	0.86	0.74	0.30
CA 15-3 (U/mL)	17.3	16.2	16.8	0.70
CA 125 (U/mL)	12.3	11.5	11.9	0.89
hPLAP (mU/L)	3.99^#^	11.12^#^	7.56	0.046
BTA (U/mL)	8.55**^,##^	3.54^##^	1.07	0.0008
IGF-I (ng/mL)	141	149	163	0.17
sIL-2R (U/mL)	364	331	352	0.75
PDGF-BB (pg/mL)	3,256**^,#^	2,587*^,#^	1,752	< 0.0001

Values shown are adjusted geometric means after correction through ANCOVA analysis for age; BMI; level of education; smoking status; total number of pregnancies; total number of months of breast-feeding; serum levels of total fat, total cholesterol, triglycerides, selenium, vitamin A, and vitamin E; and all available food-intake parameters except those related to consumption of locally grown food (in total, 21 independent variables). Correction through ANCOVA analysis for only the most relevant potential confounding factors (age, level of education, smoking status, and parameters selected for each TAP through forward stepwise regression) gave similar results, with differences concerning c-erbB-2, hPLAP, BTA, and PDGF-BB statistically significant in ANCOVA (data not shown).

a Significant differences in a Fisher’s PLSD test after correction for the above-mentioned 21 covariates, between urban areas and Peer (*p < 0.0045, and **p < 0.0001), and between Wilrijk and Hoboken (^#^*p* < 0.05, and ^##^*p* < 0.0045).

b If correction for multiple testing is implemented, statistical significance requires *p* ≤0.0045.

**Table 4:** Associations in multiple regression between levels of TAPs and parameters of internal exposure.

TAP	Parameter of internal exposure	Regression coefficient	Standardized regression coefficient (95% CI)	Squared semipartial correlation	*p*-Value^a^
Anti-p53 (index)	*I*_ex_	0.35	0.23 (0.073–0.40)	0.041	0.0050
Square root of CEA (ng/mL)	*I*_ex_	0.16	0.19 (0.033–0.35)	0.027	0.019
Anti-p53 (index)	Blood lead (nmol/L)	0.0030	0.37 (0.21–0.52)	0.10	< 0.0001
CEA (ng/mL)	Blood lead (nmol/L)	0.0021	0.18 (0.03–0.34)	0.026	0.019
TPS (U/L)	Blood lead (nmol/L)	0.107	0.22 (0.07–0.37)	0.036	0.0055
*I*_TAP_	Blood lead (nmol/L)	0.001	0.29	0.063	0.00046
c-erbB-2 (pmol/mL)	Serum zinc (μmol/L)	0.072	0.19 (0.04–0.34)	0.024	0.015
TPS (U/L)	Serum zinc (μmol/L)	8.8	0.28 (0.12–0.44)	0.053	0.00078
Ln of CA 125 (U/mL)	pg TEQ/g fat	0.0042	0.19 (0.04–0.35)	0.029	0.016
Ln of CA 125 (U/mL)	Serum copper (μmol/L)	0.022	0.16 (0.007–0.32)	0.021	0.041
Square root of c-erbB-2 (pmol/mL)	Serum copper (μmol/L)	−0.014	−0.37 (−0.50 to −0.23)	0.11	< 0.0001
Ln of IGF-I (ng/mL)	Serum copper (μmol/L)	−0.027	−0.32 (−0.47 to −0.18)	0.083	< 0.0001

CI, confidence interval. A multiple regression was performed with each TAP as the dependent variable and with all 29 available personal, lifestyle, and internal exposure parameters as independent variables. When considering association with *I*_ex_, we performed a multiple regression with each TAP as the dependent variable and with *I*_ex_ and all 21 available personal and lifestyle parameters as independent variables. Multiple regressions using as independent variables only age and the parameters (selected for each TAP through forward stepwise regression) showing the strongest association with the TAP used as the dependent variable gave similar results, with the same associations showing up as statistically significant (data not shown).

a If correction for multiple testing is implemented, statistical significance requires *p* < 0.0063.

## References

[b1-ehp0114-000887] Aickin M, Gensler H (1996). Adjusting for multiple testing when reporting research results: the Bonferroni vs Holm methods. Am J Public Health.

[b2-ehp0114-000887] Anttila A, Heikkila P, Pukkala E, Nykyri E, Kauppinen T, Hernberg S (1995). Excess lung cancer among workers exposed to lead. Scand J Work Environ Health.

[b3-ehp0114-000887] Besso A, Nyberg F, Pershagen G (2003). Air pollution and lung cancer mortality in the vicinity of a nonferrous metal smelter in Sweden. Int J Cancer.

[b4-ehp0114-000887] Bohlke K, Cramer DW, Trichopoulos D, Mantzoros CS (1998). Insulin-like growth factor-I in relation to premenopausal ductal carcinoma in situ of the breast. Epidemiology.

[b5-ehp0114-000887] Brandt-Rauf PW, Pincus MR (1998). Molecular markers of carcinogenesis. Pharmacol Ther.

[b6-ehp0114-000887] Covaci A, Koppen G, Van Cleuvenbergen R, Schepens P, Winneke G, Schoeters G (2001). Persistent organochlorine compounds in human serum of 50–65 years old women living in two regions of Flanders, Belgium. Organohalogen Compounds.

[b7-ehp0114-000887] Czene K, Lichtenstein P, Hemminki K (2002). Environmental and heritable causes of cancer among 9.6 million individuals in the Swedish Family-Cancer Database. Int J Cancer.

[b8-ehp0114-000887] Du Four VA, van Larebeke N, Janssen CR (2004). Genotoxic and mutagenic potency of environmental air samples in Flanders, Belgium. Mutat Res.

[b9-ehp0114-000887] European Commission. 2003. Directorate C—Public Health and Risk Assessment C7—Risk assessment. C7/GF/csteeop/zincs/100903 D(03). Brussels:European Commission Health and Consumer Protection Directorate-General.

[b10-ehp0114-000887] Giovannuci E (1999). Insulin-like growth factor-I and binding protein-3 and risk of cancer. Horm Res.

[b11-ehp0114-000887] Hankinson SE, Willett WC, Colditz GA, Hunter DJ, Michaud DS, Deroo B (1998). Circulating concentrations of insulin-like growth factor-I and risk of breast cancer. Lancet.

[b12-ehp0114-000887] Husgafvel-Pursiainen K, Kannio A, Oksa P, Suitiala T, Koskinen H, Partanen R (1997). Mutations, tissue accumulations, and serum levels of p53 in patients with occupational cancers from asbestos and silica exposure. Environ Mol Mutagen.

[b13-ehp0114-000887] IARC. 1987. Overall Evaluations of Carcinogenicity: An Updating of IARC Monographs Volumes 1 to 42. IARC Monogr Eval Carcinog Risk Chem Hum (suppl 7). 3482203

[b14-ehp0114-000887] IARC (1997). Summary of data reported and evaluation. IARC Monogr Eval Carcinog Risks Hum.

[b15-ehp0114-000887] Ito K, Raaijmakers R, Roobol M, Wildhagen M, Yamanaka H, Schroder FH (2005). Prostate carcinoma detection and increased prostate-specific antigen levels after 4 years in Dutch and Japanese males who had no evidence of disease at initial screening. Cancer.

[b16-ehp0114-000887] Ito K, Yamamoto T, Ohi M, Takechi H, Kurokawa K, Suzuki K (2003). Possibility of re-screening intervals of more than one year in men with PSA levels of 4.0 ng/ml or less. Prostate.

[b17-ehp0114-000887] Jacobs IJ, Skates S, Davies AP, Woolas RP, Jeyerajah A, Weidemann P (1996). Risk of diagnosis of ovarian cancer after raised serum CA 125 concentration: a prospective cohort study. BMJ.

[b18-ehp0114-000887] Jemal A, Graubard BI, Devesa SS, Flegal KM (2002). The association of blood lead level and cancer mortality among whites in the United States. Environ Health Perspect.

[b19-ehp0114-000887] Jeyarajah AR, Ind TE, Skates S, Oram DH, Jacobs IJ (1999). Serum CA125 elevation and risk of clinical detection of cancer in asymptomatic postmenopausal women. Cancer.

[b20-ehp0114-000887] Kobayashi T, Kawakubo T (1994). Prospective investigation of tumor markers and risk assessment in early cancer screening. Cancer.

[b21-ehp0114-000887] Koninckx PR, Braet P, Kennedy SH, Barlow DH (1994). Dioxin pollution and endometriosis in Belgium. Hum Reprod.

[b22-ehp0114-000887] Koppen G, Covaci A, Van Cleuvenbergen R, Schepens P, Winneke G, Nelen V (2001). Comparison of CALUX-Teq values with PCB and PCDD/PCDF measurements in human serum of the Flanders Environmental and Health Study (FLEHS). Toxicol Lett.

[b23-ehp0114-000887] Koshida K, Stigbrand T, Munck-Wikland E, Hisazumi H, Wahren B (1990). Analysis of serum placental alkaline phosphatase activity in testicular cancer and cigarette smokers. Urol Res.

[b24-ehp0114-000887] Krajewska B, Lutz W, Pilacik B (1998). Determination of blood serum oncoprotein NEU and antioncoprotein p-53 molecular biomarkers in various types of occupational exposure. Int J Occup Med Environ Health.

[b25-ehp0114-000887] Luo JC, Liu HT, Cheng TJ, Du CL, Wang JD (1999). Plasma p53 protein and anti-p53 antibody expression in vinyl chloride monomer workers in Taiwan. J Occup Environ Med.

[b26-ehp0114-000887] Lutz W, Krajewska B, Pilacik B (1997). Determination of tissue polypeptide antigens (TPA) and carcinoembryonic antigen (CEA) in serum: its value in the preliminary cancer risk assessment in asbestos exposed workers. Int J Occup Med Environ Health.

[b27-ehp0114-000887] Mayani A, Barel S, Soback S, Almagor M (1997). Dioxin concentrations in women with endometriosis. Hum Reprod.

[b28-ehp0114-000887] Milieu en Gezondheid. 2006. Milieu en Gezondheid Homepage. Available: http://www.milieu-en-gezondheid.be [accessed 24 April 2006].

[b29-ehp0114-000887] Nawrot TS, Staessen JA, Den Hond EM, Koppen G, Schoeters G, Fagard R (2002). Host and environmental determinants of polychlorinated aromatic hydrocarbons in serum of adolescents. Environ Health Perspect.

[b30-ehp0114-000887] Oka H, Tamori A, Kuroki T, Kobayashi K, Yamamoto S (1994). Prospective study of alpha-fetoprotein in cirrhotic patients monitored for development of hepatocellular carcinoma. Hepatology.

[b31-ehp0114-000887] Riboli E, Slimani N, Kaaks R (1996). Identifiability of food components for cancer chemoprevention. IARC Sci Publ.

[b32-ehp0114-000887] Rier SE, Martin DC, Bowman RE, Becker JL (1995). Immuno-responsiveness in endometriosis: implications of estrogenic toxicants. Environ Health Perspect.

[b33-ehp0114-000887] Rier SE, Martin DC, Bowman RE, Dmowski WP, Becker JL (1993). Endometriosis in rhesus monkeys (*Macaca mulatta*) following chronic exposure to 2,3,7,8-tetrachlorodibenzo-*p*-dioxin. Fundam Appl Toxicol.

[b34-ehp0114-000887] Rothman KJ 1986. Modern Epidemiology. Boston:Little, Brown & Co.

[b35-ehp0114-000887] Silbergeld E, Waalke M, Rice J (2000). Lead as a carcinogen: experimental evidence and mechanisms of action. Am J Ind Med.

[b36-ehp0114-000887] Somigliana E, Viganòò P, Tirelli AS, Felicetta I, Torresani E, Vignali M (2004). Use of the concomitant serum dosage of CA 125, CA 19-9 and interleukin-6 to detect the presence of endometriosis. Results from a series of reproductive age women undergoing laparoscopic surgery for benign gynaecological conditions. Hum Reprod.

[b37-ehp0114-000887] Staessen JA, Nawrot T, Den Hond ED, Thijs L, Fagard R, Hoppenbrouwers K (2001). Renal function, cytogenetic measurements, and sexual development in adolescents in relation to environmental pollutants: a feasibility study of biomarkers. Lancet.

[b38-ehp0114-000887] Theophanides T, Anastassopoulou J (2002). Copper and carcinogenesis. Crit Rev Oncol Hematol.

[b39-ehp0114-000887] Van Den Heuvel RL, Koppen G, Staessen JA, Den Hond E, Verheyen G, Nawrot TS (2002). Immunologic biomarkers in relation to exposure markers of PCBs and dioxins in Flemish adolescents (Belgium). Environ Health Perspect.

[b40-ehp0114-000887] van Larebeke N, Koppen G, Nelen V, Schoeters G, Van Loon H, Albering H (2004). Differences in HPRT mutant frequency among middle-aged Flemish women in association with area of residence and blood lead levels. Biomarkers.

[b41-ehp0114-000887] Verkasalo PK, Kaprio J, Koskenvuo M, Pukkala E (1999). Genetic predisposition, environment and cancer incidence: a nationwide twin study in Finland, 1976–1995. Int J Cancer.

[b42-ehp0114-000887] Wolk A, Mantzoros CS, Andersson SO, Bergstrom R, Signorello LB, Lagiou P (1998). Insulin-like growth factor 1 and prostate cancer risk: a population-based, case-control study. J Natl Cancer Inst.

[b43-ehp0114-000887] Wong RH, Du CL, Wang JD, Chan CC, Luo JC, Cheng TJ (2002). XRCC1 and CYP2E1 polymorphisms as susceptibility factors of plasma mutant p53 protein and anti-p53 antibody expression in vinyl chloride monomer-exposed polyvinyl chloride workers. Cancer Epidemiol Biomarkers Prev.

